# First person – Rajeshwari B. R.

**DOI:** 10.1242/bio.059944

**Published:** 2023-05-04

## Abstract

First Person is a series of interviews with the first authors of a selection of papers published in Biology Open, helping researchers promote themselves alongside their papers. Rajeshwari B. R. is first author on ‘ Kinetics of Arf1 inactivation regulates Golgi organization and function in non-adherent fibroblasts’, published in BiO. Rajeshwari is a PhD student in the lab of Dr Nagaraj Balasubramanian at the Indian Institute of Science Education and Research (IISER), Pune, investigating the role of cell-matrix adhesion in regulating the Golgi organization and function.


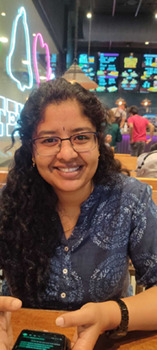


**Rajeshwari B. R.**

**Describe your scientific journey and your current research focus**

Presently, I am pursuing my PhD in Dr Nagaraj Balasubramanian's laboratory, where our research is centered around the regulation of various cellular signaling pathways by adhesion. Specifically, my research project aims to unravel the impact of cell-matrix adhesion on the organization and functionality of distinct Golgi compartments. Our key focus is on examining the adhesion-dependent regulation of the Golgi organization and using it to understand the role of small GTPase Arf1 in modulating the organization and function of the Golgi.

**Who or what inspired you to become a scientist?**

It wasn't a single person or moment that sparked my interest in research. Rather, I was fortunate enough to have inspiring teachers and professors who influenced me throughout my academic journey. Their encouragement and motivation at various stages led me to pursue advanced studies in biological sciences.

**How would you explain the main finding of your paper?**

The Golgi apparatus is a critical player in regulating various cellular processes, including the processing and trafficking of lipids and proteins within the cell. Proper organization of the Golgi is crucial for its optimal functioning, as changes in its organization can significantly alter its functions and have been associated with diseases like neurodegeneration and cancer. Integrin-dependent cell-matrix adhesion is one of several signaling pathways that can contribute to the organization of the Golgi. In mammalian cells, the Golgi is arranged in a disk-like ribbon structure around the MTOC. Loss of cell-matrix adhesion results in the disorganization of the Golgi compartments. Interestingly, it leads to greater disorganization of the trans-Golgi compartment compared to the cis-medial-Golgi. Our research indicates that this difference in Golgi disorganization is attributed to differences in Arf1 activation at the cis-medial and trans-Golgi in non-adherent cells. In non-adherent cells, the cis-Golgi retains active Arf1, whereas the trans-Golgi does not. Further, inhibition of Arf1 activation in non-adherent fibroblasts leads to cis-medial Golgi fragmentation and fusion with the ER. Our findings demonstrate that the extent of cis-medial Golgi fragmentation in non-adherent fibroblasts depends on both the final levels of active Arf1 and the kinetics of the drop in active Arf1 levels, as determined through the use of pharmacological inhibitors.

**What are the potential implications of this finding for your field of research?**

Altered organization of the Golgi has been associated with normal cell division and various diseases, including cancer. However, there is limited understanding regarding the regulation of changes in Golgi organization and how they contribute to disease progression. Our investigations on non-adherent fibroblasts reveal how changes in active Arf1 levels at the Golgi and the kinetics of such changes can have diverse impacts on Golgi organization and function. These findings indicate that precise regulation of Arf1 activity at the Golgi could enable cells to modify Golgi organization in distinct ways to affect cell function. How this is used in normal and disease conditions needs further exploration.

“Our investigations on non-adherent fibroblasts reveal how changes in active Arf1 levels at the Golgi and the kinetics of such changes can have diverse impacts on Golgi organization and function.”

**Figure:**
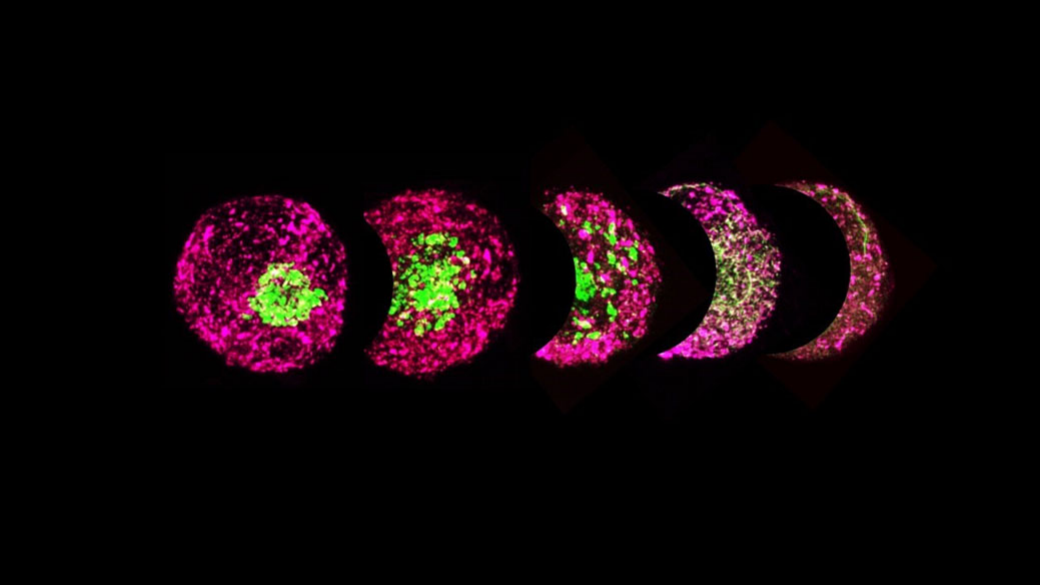
Fragmentation of cis-medial Golgi (ManII-GFP) and its fall back to ER (ss-KDEL-RFP) when WT-MEFs suspended for 60 min were treated for 30 min with increasing concentration of BFA.

**Which part of this research project was the most rewarding?**

Microscopy has been an enjoyable aspect of my work, particularly being able to use fluorescently tagged Golgi markers to observe changes in Golgi organization. It felt great visualizing the localization of active Arf1 in relation to the cis- and trans-Golgi compartments in non-adherent cells.

**What do you enjoy most about being an early-career researcher**?****

Every day, I have the privilege of gaining new knowledge and insights, which makes my journey all the more thrilling. I get to learn a great deal from interacting with researchers from diverse fields.

**What piece of advice would you give to the next generation of researchers?**

As you move forward, prioritize the acquisition of new skills. The skills we cultivate now hold greater significance for the future than the outcomes we achieve. Additionally, networking is key. Seize every opportunity to present your research and listen to others’ presentations, even if they aren't from your field. Sometimes, valuable insights come from unexpected sources.

“Sometimes, valuable insights come from unexpected sources.”

**What's next for you?**

My thesis defence is fast approaching. Post that I am eagerly anticipating the opportunity to make a meaningful contribution to the ongoing research in life sciences.
